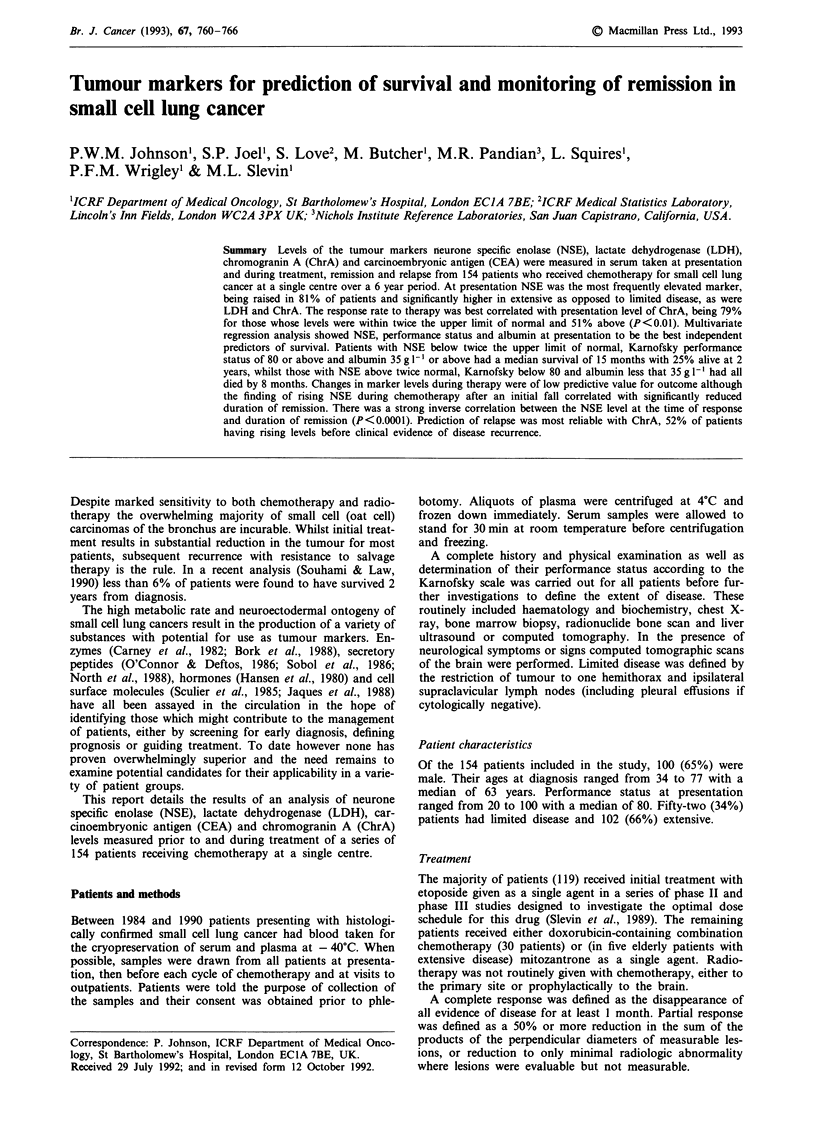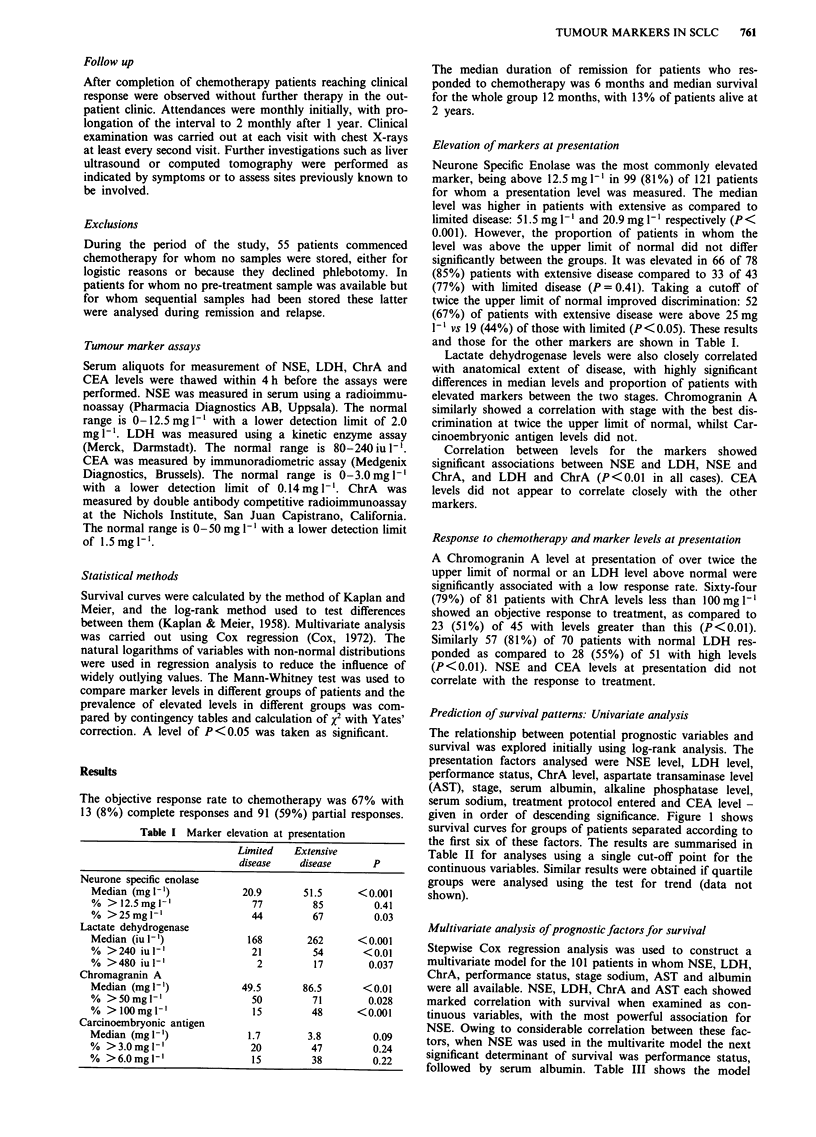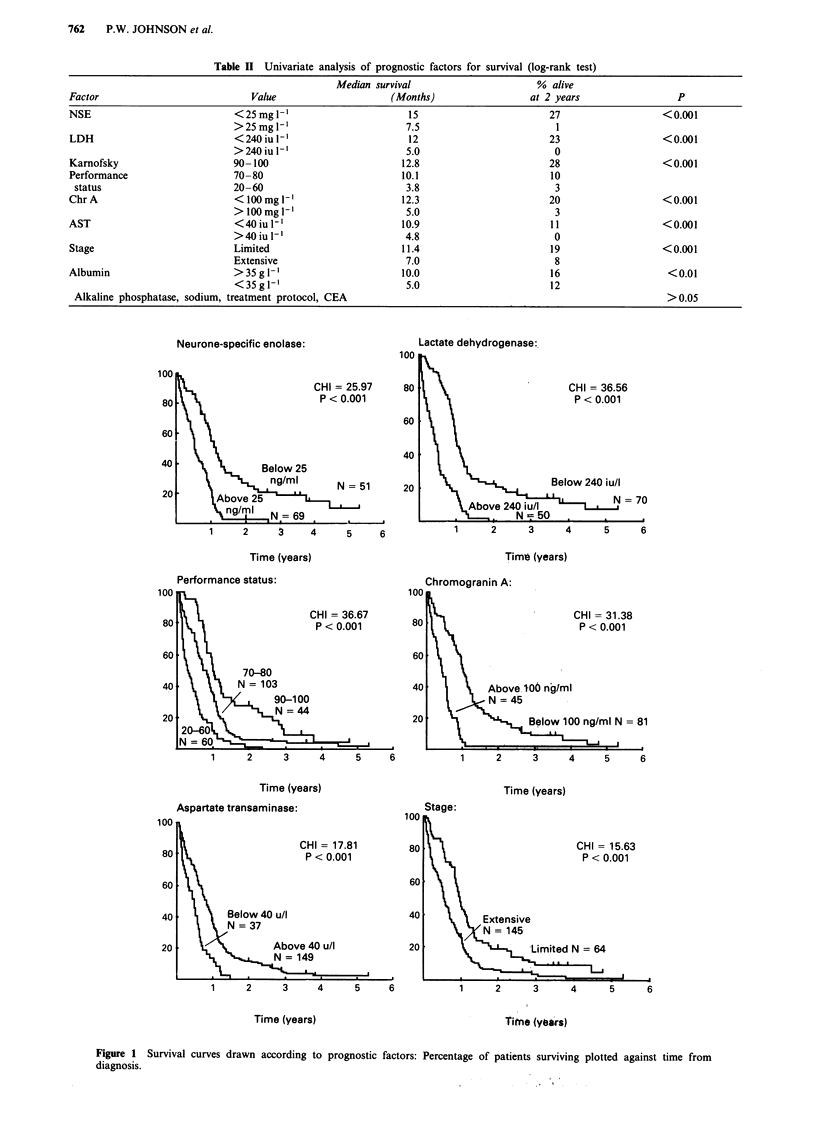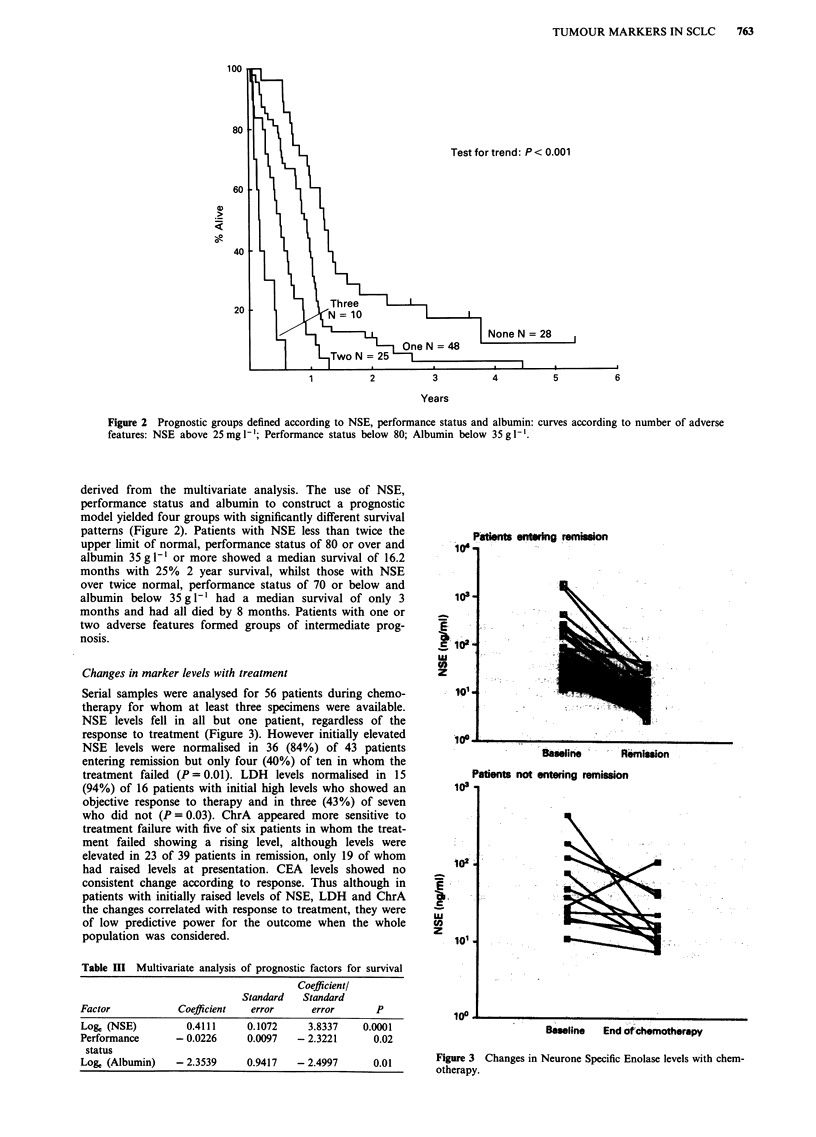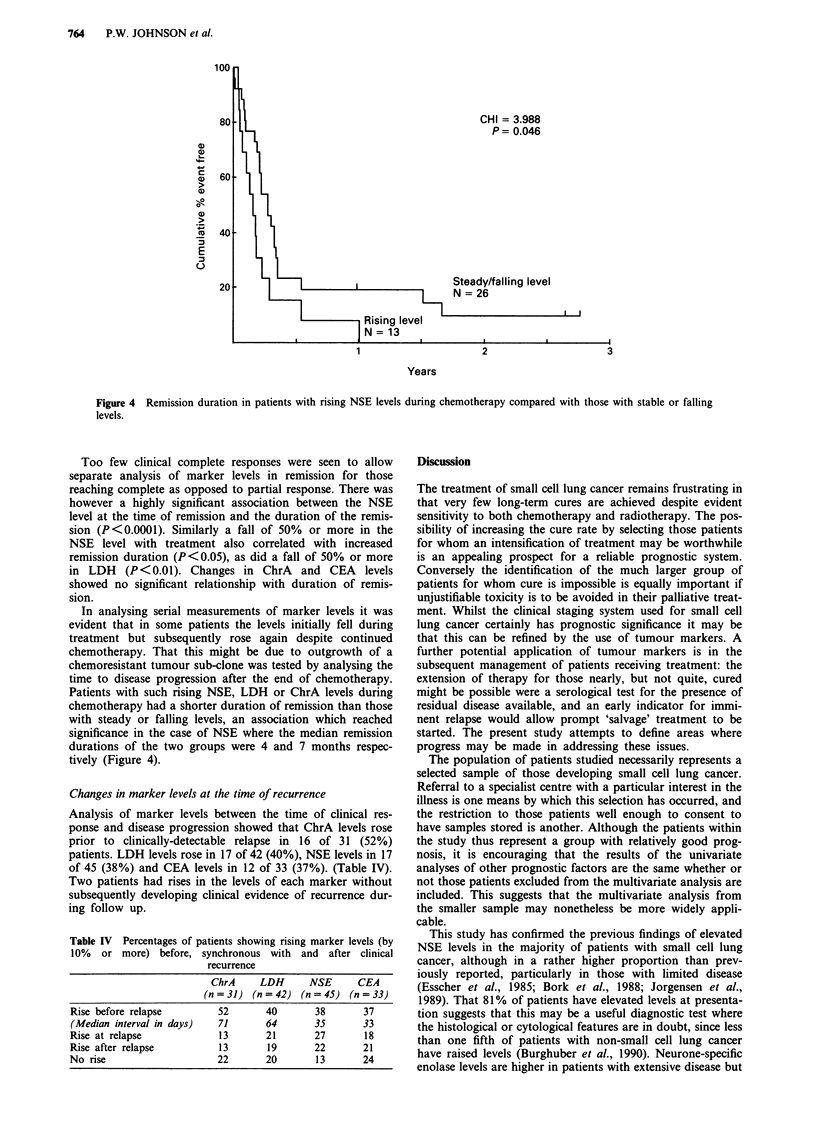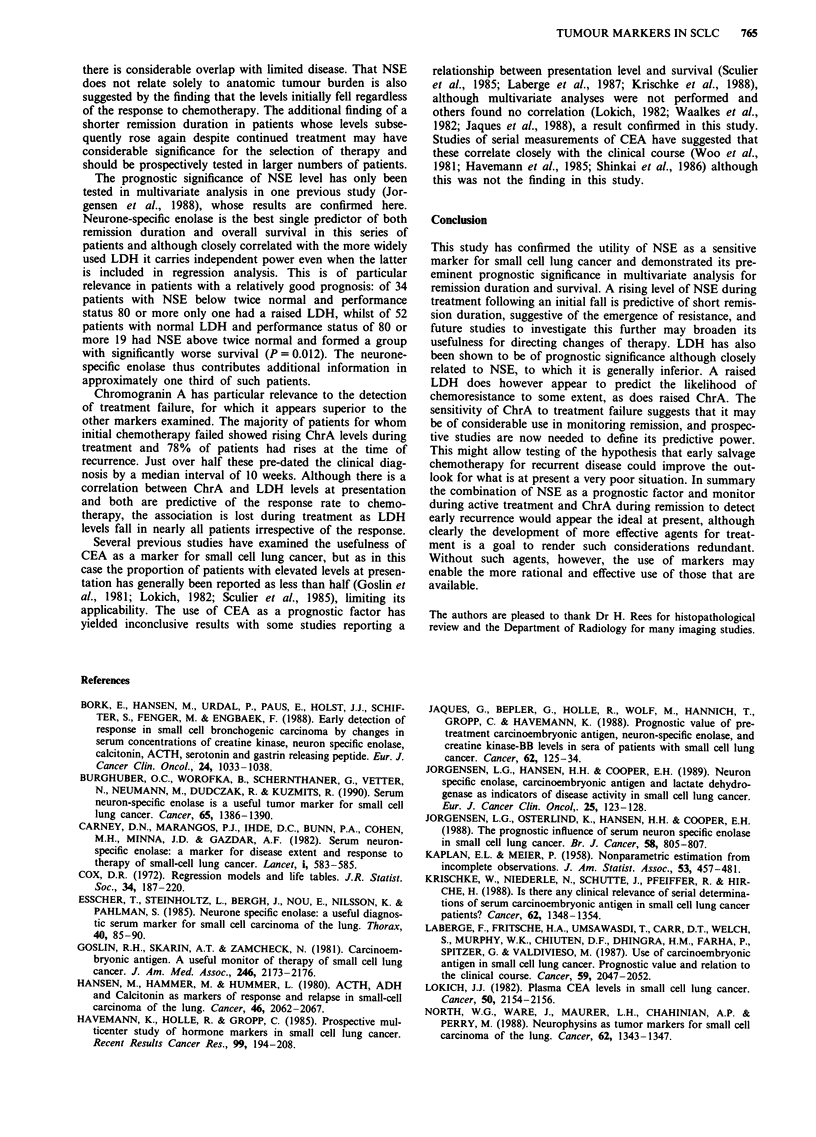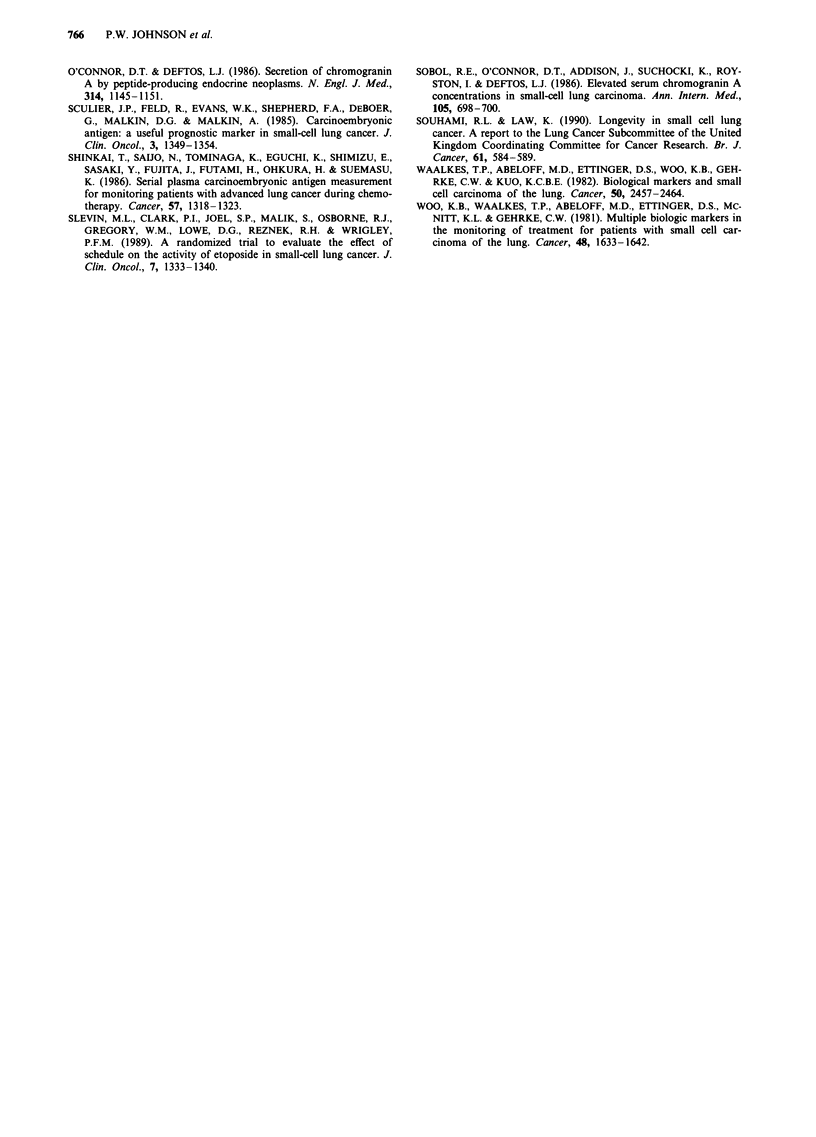# Tumour markers for prediction of survival and monitoring of remission in small cell lung cancer.

**DOI:** 10.1038/bjc.1993.138

**Published:** 1993-04

**Authors:** P. W. Johnson, S. P. Joel, S. Love, M. Butcher, M. R. Pandian, L. Squires, P. F. Wrigley, M. L. Slevin

**Affiliations:** ICRF Department of Medical Oncology, St Bartholomew's Hospital, London, UK.

## Abstract

Levels of the tumour markers neurone specific enolase (NSE), lactate dehydrogenase (LDH), chromogranin A (ChrA) and carcinoembryonic antigen (CEA) were measured in serum taken at presentation and during treatment, remission and relapse from 154 patients who received chemotherapy for small cell lung cancer at a single centre over a 6 year period. At presentation NSE was the most frequently elevated marker, being raised in 81% of patients and significantly higher in extensive as opposed to limited disease, as were LDH and ChrA. The response rate to therapy was best correlated with presentation level of ChrA, being 79% for those whose levels were within twice the upper limit of normal and 51% above (P < 0.01). Multivariate regression analysis showed NSE, performance status and albumin at presentation to be the best independent predictors of survival. Patients with NSE below twice the upper limit of normal, Karnofsky performance status of 80 or above and albumin 35 g l-1 or above had a median survival of 15 months with 25% alive at 2 years, whilst those with NSE above twice normal, Karnofsky below 80 and albumin less that 35 g l-1 had all died by 8 months. Changes in marker levels during therapy were of low predictive value for outcome although the finding of rising NSE during chemotherapy after an initial fall correlated with significantly reduced duration of remission. There was a strong inverse correlation between the NSE level at the time of response and duration of remission (P < 0.0001). Prediction of relapse was most reliable with ChrA, 52% of patients having rising levels before clinical evidence of disease recurrence.


Br. J. Cancer (1993), 67, 760-766 © Macmillan Press Ltd., 1993

### Tumour markers for prediction of survival and monitoring of remission in small cell lung cancer

P.W.M. Johnson[1], S.P. Joel[1], S. Love[2], M. Butcher[1], M.R. Pandian[3], L. Squires[1], P.F.M. Wrigley[1] & M.L. Slevin[1]

[1]*ICRF Department of Medical Oncology, St Bartholomew's Hospital, London EC1A 7BE;* [2]*ICRF Medical Statistics Laboratory, Lincoln's Inn Fields, London WC2A 3PX UK;* [3]*Nichols Institute Reference Laboratories, San Juan Capistrano, California, USA.*

**Summary** Levels of the tumour markers neurone specific enolase (NSE), lactate dehydrogenase (LDH), chromogranin A (ChrA) and carcinoembryonic antigen (CEA) were measured in serum taken at presentation and during treatment, remission and relapse from 154 patients who received chemotherapy for small cell lung cancer at a single centre over a 6 year period. At presentation NSE was the most frequently elevated marker, being raised in 81% of patients and significantly higher in extensive as opposed to limited disease, as were LDH and ChrA. The response rate to therapy was best correlated with presentation level of ChrA, being 79% for those whose levels were within twice the upper limit of normal and 51% above ($P<0.01$). Multivariate regression analysis showed NSE, performance status and albumin at presentation to be the best independent predictors of survival. Patients with NSE below twice the upper limit of normal, Karnofsky performance status of 80 or above and albumin 35 g $l^{-1}$ or above had a median survival of 15 months with 25% alive at 2 years, whilst those with NSE above twice normal, Karnofsky below 80 and albumin less that 35 g $l^{-1}$ had all died by 8 months. Changes in marker levels during therapy were of low predictive value for outcome although the finding of rising NSE during chemotherapy after an initial fall correlated with significantly reduced duration of remission. There was a strong inverse correlation between the NSE level at the time of response and duration of remission ($P<0.0001$). Prediction of relapse was most reliable with ChrA, 52% of patients having rising levels before clinical evidence of disease recurrence.

Despite marked sensitivity to both chemotherapy and radiotherapy the overwhelming majority of small cell (oat cell) carcinomas of the bronchus are incurable. Whilst initial treatment results in substantial reduction in the tumour for most patients, subsequent recurrence with resistance to salvage therapy is the rule. In a recent analysis (Souhami & Law, 1990) less than 6% of patients were found to have survived 2 years from diagnosis.

The high metabolic rate and neuroectodermal ontogeny of small cell lung cancers result in the production of a variety of substances with potential for use as tumour markers. Enzymes (Carney *et al.*, 1982; Bork *et al.*, 1988), secretory peptides (O'Connor & Deftos, 1986; Sobol *et al.*, 1986; North *et al.*, 1988), hormones (Hansen *et al.*, 1980) and cell surface molecules (Sculier *et al.*, 1985; Jaques *et al.*, 1988) have all been assayed in the circulation in the hope of identifying those which might contribute to the management of patients, either by screening for early diagnosis, defining prognosis or guiding treatment. To date however none has proven overwhelmingly superior and the need remains to examine potential candidates for their applicability in a variety of patient groups.

This report details the results of an analysis of neurone specific enolase (NSE), lactate dehydrogenase (LDH), carcinoembryonic antigen (CEA) and chromogranin A (ChrA) levels measured prior to and during treatment of a series of 154 patients receiving chemotherapy at a single centre.

### Patients and methods

Between 1984 and 1990 patients presenting with histologically confirmed small cell lung cancer had blood taken for the cryopreservation of serum and plasma at $-40°C$. When possible, samples were drawn from all patients at presentation, then before each cycle of chemotherapy and at visits to outpatients. Patients were told the purpose of collection of the samples and their consent was obtained prior to phlebotomy. Aliquots of plasma were centrifuged at 4°C and frozen down immediately. Serum samples were allowed to stand for 30 min at room temperature before centrifugation and freezing.

A complete history and physical examination as well as determination of their performance status according to the Karnofsky scale was carried out for all patients before further investigations to define the extent of disease. These routinely included haematology and biochemistry, chest X-ray, bone marrow biopsy, radionuclide bone scan and liver ultrasound or computed tomography. In the presence of neurological symptoms or signs computed tomographic scans of the brain were performed. Limited disease was defined by the restriction of tumour to one hemithorax and ipsilateral supraclavicular lymph nodes (including pleural effusions if cytologically negative).

### *Patient characteristics*

Of the 154 patients included in the study, 100 (65%) were male. Their ages at diagnosis ranged from 34 to 77 with a median of 63 years. Performance status at presentation ranged from 20 to 100 with a median of 80. Fifty-two (34%) patients had limited disease and 102 (66%) extensive.

### *Treatment*

The majority of patients (119) received initial treatment with etoposide given as a single agent in a series of phase II and phase III studies designed to investigate the optimal dose schedule for this drug (Slevin *et al.*, 1989). The remaining patients received either doxorubicin-containing combination chemotherapy (30 patients) or (in five elderly patients with extensive disease) mitozantrone as a single agent. Radiotherapy was not routinely given with chemotherapy, either to the primary site or prophylactically to the brain.

A complete response was defined as the disappearance of all evidence of disease for at least 1 month. Partial response was defined as a 50% or more reduction in the sum of the products of the perpendicular diameters of measurable lesions, or reduction to only minimal radiologic abnormality where lesions were evaluable but not measurable.

Correspondence: P. Johnson, ICRF Department of Medical Oncology, St Bartholomew's Hospital, London EC1A 7BE, UK.
Received 29 July 1992; and in revised form 12 October 1992.

*Follow up*

After completion of chemotherapy patients reaching clinical response were observed without further therapy in the out-patient clinic. Attendances were monthly initially, with prolongation of the interval to 2 monthly after 1 year. Clinical examination was carried out at each visit with chest X-rays at least every second visit. Further investigations such as liver ultrasound or computed tomography were performed as indicated by symptoms or to assess sites previously known to be involved.

*Exclusions*

During the period of the study, 55 patients commenced chemotherapy for whom no samples were stored, either for logistic reasons or because they declined phlebotomy. In patients for whom no pre-treatment sample was available but for whom sequential samples had been stored these latter were analysed during remission and relapse.

*Tumour marker assays*

Serum aliquots for measurement of NSE, LDH, ChrA and CEA levels were thawed within 4 h before the assays were performed. NSE was measured in serum using a radioimmunoassay (Pharmacia Diagnostics AB, Uppsala). The normal range is 0–12.5 mg $l^{-1}$ with a lower detection limit of 2.0 mg $l^{-1}$. LDH was measured using a kinetic enzyme assay (Merck, Darmstadt). The normal range is 80–240 iu $l^{-1}$. CEA was measured by immunoradiometric assay (Medgenix Diagnostics, Brussels). The normal range is 0–3.0 mg $l^{-1}$ with a lower detection limit of 0.14 mg $l^{-1}$. ChrA was measured by double antibody competitive radioimmunoassay at the Nichols Institute, San Juan Capistrano, California. The normal range is 0–50 mg $l^{-1}$ with a lower detection limit of 1.5 mg $l^{-1}$.

*Statistical methods*

Survival curves were calculated by the method of Kaplan and Meier, and the log-rank method used to test differences between them (Kaplan & Meier, 1958). Multivariate analysis was carried out using Cox regression (Cox, 1972). The natural logarithms of variables with non-normal distributions were used in regression analysis to reduce the influence of widely outlying values. The Mann-Whitney test was used to compare marker levels in different groups of patients and the prevalence of elevated levels in different groups was compared by contingency tables and calculation of $\chi^2$ with Yates' correction. A level of $P<0.05$ was taken as significant.

### Results

The objective response rate to chemotherapy was 67% with 13 (8%) complete responses and 91 (59%) partial responses.

**Table I** Marker elevation at presentation

| | *Limited disease* | *Extensive disease* | *P* |
|---|---|---|---|
| Neurone specific enolase | | | |
| Median (mg $l^{-1}$) | 20.9 | 51.5 | <0.001 |
| % >12.5 mg $l^{-1}$ | 77 | 85 | 0.41 |
| % >25 mg $l^{-1}$ | 44 | 67 | 0.03 |
| Lactate dehydrogenase | | | |
| Median (iu $l^{-1}$) | 168 | 262 | <0.001 |
| % >240 iu $l^{-1}$ | 21 | 54 | <0.01 |
| % >480 iu $l^{-1}$ | 2 | 17 | 0.037 |
| Chromagranin A | | | |
| Median (mg $l^{-1}$) | 49.5 | 86.5 | <0.01 |
| % >50 mg $l^{-1}$ | 50 | 71 | 0.028 |
| % >100 mg $l^{-1}$ | 15 | 48 | <0.001 |
| Carcinoembryonic antigen | | | |
| Median (mg $l^{-1}$) | 1.7 | 3.8 | 0.09 |
| % >3.0 mg $l^{-1}$ | 20 | 47 | 0.24 |
| % >6.0 mg $l^{-1}$ | 15 | 38 | 0.22 |

The median duration of remission for patients who responded to chemotherapy was 6 months and median survival for the whole group 12 months, with 13% of patients alive at 2 years.

*Elevation of markers at presentation*

Neurone Specific Enolase was the most commonly elevated marker, being above 12.5 mg $l^{-1}$ in 99 (81%) of 121 patients for whom a presentation level was measured. The median level was higher in patients with extensive as compared to limited disease: 51.5 mg $l^{-1}$ and 20.9 mg $l^{-1}$ respectively ($P<$ 0.001). However, the proportion of patients in whom the level was above the upper limit of normal did not differ significantly between the groups. It was elevated in 66 of 78 (85%) patients with extensive disease compared to 33 of 43 (77%) with limited disease ($P=0.41$). Taking a cutoff of twice the upper limit of normal improved discrimination: 52 (67%) of patients with extensive disease were above 25 mg $l^{-1}$ *vs* 19 (44%) of those with limited ($P<0.05$). These results and those for the other markers are shown in Table I.

Lactate dehydrogenase levels were also closely correlated with anatomical extent of disease, with highly significant differences in median levels and proportion of patients with elevated markers between the two stages. Chromogranin A similarly showed a correlation with stage with the best discrimination at twice the upper limit of normal, whilst Carcinoembryonic antigen levels did not.

Correlation between levels for the markers showed significant associations between NSE and LDH, NSE and ChrA, and LDH and ChrA ($P<0.01$ in all cases). CEA levels did not appear to correlate closely with the other markers.

*Response to chemotherapy and marker levels at presentation*

A Chromogranin A level at presentation of over twice the upper limit of normal or an LDH level above normal were significantly associated with a low response rate. Sixty-four (79%) of 81 patients with ChrA levels less than 100 mg $l^{-1}$ showed an objective response to treatment, as compared to 23 (51%) of 45 with levels greater than this ($P<0.01$). Similarly 57 (81%) of 70 patients with normal LDH responded as compared to 28 (55%) of 51 with high levels ($P<0.01$). NSE and CEA levels at presentation did not correlate with the response to treatment.

*Prediction of survival patterns: Univariate analysis*

The relationship between potential prognostic variables and survival was explored initially using log-rank analysis. The presentation factors analysed were NSE level, LDH level, performance status, ChrA level, aspartate transaminase level (AST), stage, serum albumin, alkaline phosphatase level, serum sodium, treatment protocol entered and CEA level – given in order of descending significance. Figure 1 shows survival curves for groups of patients separated according to the first six of these factors. The results are summarised in Table II for analyses using a single cut-off point for the continuous variables. Similar results were obtained if quartile groups were analysed using the test for trend (data not shown).

*Multivariate analysis of prognostic factors for survival*

Stepwise Cox regression analysis was used to construct a multivariate model for the 101 patients in whom NSE, LDH, ChrA, performance status, stage sodium, AST and albumin were all available. NSE, LDH, ChrA and AST each showed marked correlation with survival when examined as continuous variables, with the most powerful association for NSE. Owing to considerable correlation between these factors, when NSE was used in the multivarite model the next significant determinant of survival was performance status, followed by serum albumin. Table III shows the model

**Table II** Univariate analysis of prognostic factors for survival (log-rank test)

| *Factor* | *Value* | *Median survival (Months)* | *% alive at 2 years* | *P* |
|---|---|---|---|---|
| NSE | $<25\ mg\ l^{-1}$ | 15 | 27 | $<0.001$ |
| | $>25\ mg\ l^{-1}$ | 7.5 | 1 | |
| LDH | $<240\ iu\ l^{-1}$ | 12 | 23 | $<0.001$ |
| | $>240\ iu\ l^{-1}$ | 5.0 | 0 | |
| Karnofsky | 90–100 | 12.8 | 28 | $<0.001$ |
| Performance | 70–80 | 10.1 | 10 | |
| status | 20–60 | 3.8 | 3 | |
| Chr A | $<100\ mg\ l^{-1}$ | 12.3 | 20 | $<0.001$ |
| | $>100\ mg\ l^{-1}$ | 5.0 | 3 | |
| AST | $<40\ iu\ l^{-1}$ | 10.9 | 11 | $<0.001$ |
| | $>40\ iu\ l^{-1}$ | 4.8 | 0 | |
| Stage | Limited | 11.4 | 19 | $<0.001$ |
| | Extensive | 7.0 | 8 | |
| Albumin | $>35\ g\ l^{-1}$ | 10.0 | 16 | $<0.01$ |
| | $<35\ g\ l^{-1}$ | 5.0 | 12 | |
| Alkaline phosphatase, sodium, treatment protocol, CEA | | | | $>0.05$ |

**Figure 1** Survival curves drawn according to prognostic factors: Percentage of patients surviving plotted against time from diagnosis.

**Figure 2** Prognostic groups defined according to NSE, performance status and albumin: curves according to number of adverse features: NSE above 25 mg $l^{-1}$; Performance status below 80; Albumin below 35 g $l^{-1}$.

derived from the multivariate analysis. The use of NSE, performance status and albumin to construct a prognostic model yielded four groups with significantly different survival patterns (Figure 2). Patients with NSE less than twice the upper limit of normal, performance status of 80 or over and albumin 35 g $l^{-1}$ or more showed a median survival of 16.2 months with 25% 2 year survival, whilst those with NSE over twice normal, performance status of 70 or below and albumin below 35 g $l^{-1}$ had a median survival of only 3 months and had all died by 8 months. Patients with one or two adverse features formed groups of intermediate prognosis.

### *Changes in marker levels with treatment*

Serial samples were analysed for 56 patients during chemotherapy for whom at least three specimens were available. NSE levels fell in all but one patient, regardless of the response to treatment (Figure 3). However initially elevated NSE levels were normalised in 36 (84%) of 43 patients entering remission but only four (40%) of ten in whom the treatment failed ($P = 0.01$). LDH levels normalised in 15 (94%) of 16 patients with initial high levels who showed an objective response to therapy and in three (43%) of seven who did not ($P = 0.03$). ChrA appeared more sensitive to treatment failure with five of six patients in whom the treatment failed showing a rising level, although levels were elevated in 23 of 39 patients in remission, only 19 of whom had raised levels at presentation. CEA levels showed no consistent change according to response. Thus although in patients with initially raised levels of NSE, LDH and ChrA the changes correlated with response to treatment, they were of low predictive power for the outcome when the whole population was considered.

**Table III** Multivariate analysis of prognostic factors for survival

| *Factor* | *Coefficient* | *Standard error* | *Coefficient/ Standard error* | *P* |
|---|---|---|---|---|
| $Log_e$ (NSE) | 0.4111 | 0.1072 | 3.8337 | 0.0001 |
| Performance status | – 0.0226 | 0.0097 | – 2.3221 | 0.02 |
| $Log_e$ (Albumin) | – 2.3539 | 0.9417 | – 2.4997 | 0.01 |

**Figure 3** Changes in Neurone Specific Enolase levels with chemotherapy.

**Figure 4** Remission duration in patients with rising NSE levels during chemotherapy compared with those with stable or falling levels.

Too few clinical complete responses were seen to allow separate analysis of marker levels in remission for those reaching complete as opposed to partial response. There was however a highly significant association between the NSE level at the time of remission and the duration of the remission ($P<0.0001$). Similarly a fall of 50% or more in the NSE level with treatment also correlated with increased remission duration ($P<0.05$), as did a fall of 50% or more in LDH ($P<0.01$). Changes in ChrA and CEA levels showed no significant relationship with duration of remission.

In analysing serial measurements of marker levels it was evident that in some patients the levels initially fell during treatment but subsequently rose again despite continued chemotherapy. That this might be due to outgrowth of a chemoresistant tumour sub-clone was tested by analysing the time to disease progression after the end of chemotherapy. Patients with such rising NSE, LDH or ChrA levels during chemotherapy had a shorter duration of remission than those with steady or falling levels, an association which reached significance in the case of NSE where the median remission durations of the two groups were 4 and 7 months respectively (Figure 4).

### *Changes in marker levels at the time of recurrence*

Analysis of marker levels between the time of clinical response and disease progression showed that ChrA levels rose prior to clinically-detectable relapse in 16 of 31 (52%) patients. LDH levels rose in 17 of 42 (40%), NSE levels in 17 of 45 (38%) and CEA levels in 12 of 33 (37%). (Table IV). Two patients had rises in the levels of each marker without subsequently developing clinical evidence of recurrence during follow up.

**Table IV** Percentages of patients showing rising marker levels (by 10% or more) before, synchronous with and after clinical recurrence

| | *ChrA (n = 31)* | *LDH (n = 42)* | *NSE (n = 45)* | *CEA (n = 33)* |
|---|---|---|---|---|
| Rise before relapse | 52 | 40 | 38 | 37 |
| *(Median interval in days)* | *71* | *64* | *35* | *33* |
| Rise at relapse | 13 | 21 | 27 | 18 |
| Rise after relapse | 13 | 19 | 22 | 21 |
| No rise | 22 | 20 | 13 | 24 |

### Discussion

The treatment of small cell lung cancer remains frustrating in that very few long-term cures are achieved despite evident sensitivity to both chemotherapy and radiotherapy. The possibility of increasing the cure rate by selecting those patients for whom an intensification of treatment may be worthwhile is an appealing prospect for a reliable prognostic system. Conversely the identification of the much larger group of patients for whom cure is impossible is equally important if unjustifiable toxicity is to be avoided in their palliative treatment. Whilst the clinical staging system used for small cell lung cancer certainly has prognostic significance it may be that this can be refined by the use of tumour markers. A further potential application of tumour markers is in the subsequent management of patients receiving treatment: the extension of therapy for those nearly, but not quite, cured might be possible were a serological test for the presence of residual disease available, and an early indicator for imminent relapse would allow prompt 'salvage' treatment to be started. The present study attempts to define areas where progress may be made in addressing these issues.

The population of patients studied necessarily represents a selected sample of those developing small cell lung cancer. Referral to a specialist centre with a particular interest in the illness is one means by which this selection has occurred, and the restriction to those patients well enough to consent to have samples stored is another. Although the patients within the study thus represent a group with relatively good prognosis, it is encouraging that the results of the univariate analyses of other prognostic factors are the same whether or not those patients excluded from the multivariate analysis are included. This suggests that the multivariate analysis from the smaller sample may nonetheless be more widely applicable.

This study has confirmed the previous findings of elevated NSE levels in the majority of patients with small cell lung cancer, although in a rather higher proportion than previously reported, particularly in those with limited disease (Esscher *et al.*, 1985; Bork *et al.*, 1988; Jorgensen *et al.*, 1989). That 81% of patients have elevated levels at presentation suggests that this may be a useful diagnostic test where the histological or cytological features are in doubt, since less than one fifth of patients with non-small cell lung cancer have raised levels (Burghuber *et al.*, 1990). Neurone-specific enolase levels are higher in patients with extensive disease but there is considerable overlap with limited disease. That NSE does not relate solely to anatomic tumour burden is also suggested by the finding that the levels initially fell regardless of the response to chemotherapy. The additional finding of a shorter remission duration in patients whose levels subsequently rose again despite continued treatment may have considerable significance for the selection of therapy and should be prospectively tested in larger numbers of patients.

The prognostic significance of NSE level has only been tested in multivariate analysis in one previous study (Jorgensen *et al.*, 1988), whose results are confirmed here. Neurone-specific enolase is the best single predictor of both remission duration and overall survival in this series of patients and although closely correlated with the more widely used LDH it carries independent power even when the latter is included in regression analysis. This is of particular relevance in patients with a relatively good prognosis: of 34 patients with NSE below twice normal and performance status 80 or more only one had a raised LDH, whilst of 52 patients with normal LDH and performance status of 80 or more 19 had NSE above twice normal and formed a group with significantly worse survival ($P = 0.012$). The neurone-specific enolase thus contributes additional information in approximately one third of such patients.

Chromogranin A has particular relevance to the detection of treatment failure, for which it appears superior to the other markers examined. The majority of patients for whom initial chemotherapy failed showed rising ChrA levels during treatment and 78% of patients had rises at the time of recurrence. Just over half these pre-dated the clinical diagnosis by a median interval of 10 weeks. Although there is a correlation between ChrA and LDH levels at presentation and both are predictive of the response rate to chemotherapy, the association is lost during treatment as LDH levels fall in nearly all patients irrespective of the response.

Several previous studies have examined the usefulness of CEA as a marker for small cell lung cancer, but as in this case the proportion of patients with elevated levels at presentation has generally been reported as less than half (Goslin *et al.*, 1981; Lokich, 1982; Sculier *et al.*, 1985), limiting its applicability. The use of CEA as a prognostic factor has yielded inconclusive results with some studies reporting a relationship between presentation level and survival (Sculier *et al.*, 1985; Laberge *et al.*, 1987; Krischke *et al.*, 1988), although multivariate analyses were not performed and others found no correlation (Lokich, 1982; Waalkes *et al.*, 1982; Jaques *et al.*, 1988), a result confirmed in this study. Studies of serial measurements of CEA have suggested that these correlate closely with the clinical course (Woo *et al.*, 1981; Havemann *et al.*, 1985; Shinkai *et al.*, 1986) although this was not the finding in this study.

### Conclusion

This study has confirmed the utility of NSE as a sensitive marker for small cell lung cancer and demonstrated its pre-eminent prognostic significance in multivariate analysis for remission duration and survival. A rising level of NSE during treatment following an initial fall is predictive of short remission duration, suggestive of the emergence of resistance, and future studies to investigate this further may broaden its usefulness for directing changes of therapy. LDH has also been shown to be of prognostic significance although closely related to NSE, to which it is generally inferior. A raised LDH does however appear to predict the likelihood of chemoresistance to some extent, as does raised ChrA. The sensitivity of ChrA to treatment failure suggests that it may be of considerable use in monitoring remission, and prospective studies are now needed to define its predictive power. This might allow testing of the hypothesis that early salvage chemotherapy for recurrent disease could improve the outlook for what is at present a very poor situation. In summary the combination of NSE as a prognostic factor and monitor during active treatment and ChrA during remission to detect early recurrence would appear the ideal at present, although clearly the development of more effective agents for treatment is a goal to render such considerations redundant. Without such agents, however, the use of markers may enable the more rational and effective use of those that are available.

The authors are pleased to thank Dr H. Rees for histopathological review and the Department of Radiology for many imaging studies.

### References

BORK, E., HANSEN, M., URDAL, P., PAUS, E., HOLST, J.J., SCHIFTER, S., FENGER, M. & ENGBAEK, F. (1988). Early detection of response in small cell bronchogenic carcinoma by changes in serum concentrations of creatine kinase, neuron specific enolase, calcitonin, ACTH, serotonin and gastrin releasing peptide. *Eur. J. Cancer Clin. Oncol.*, **24**, 1033–1038.

BURGHUBER, O.C., WOROFKA, B., SCHERNTHANER, G., VETTER, N., NEUMANN, M., DUDCZAK, R. & KUZMITS, R. (1990). Serum neuron-specific enolase is a useful tumor marker for small cell lung cancer. *Cancer*, **65**, 1386–1390.

CARNEY, D.N., MARANGOS, P.J., IHDE, D.C., BUNN, P.A., COHEN, M.H., MINNA, J.D. & GAZDAR, A.F. (1982). Serum neuron-specific enolase: a marker for disease extent and response to therapy of small-cell lung cancer. *Lancet*, **i**, 583–585.

COX, D.R. (1972). Regression models and life tables. *J.R. Statist. Soc.*, **34**, 187–220.

ESSCHER, T., STEINHOLTZ, L., BERGH, J., NOU, E., NILSSON, K. & PAHLMAN, S. (1985). Neurone specific enolase: a useful diagnostic serum marker for small cell carcinoma of the lung. *Thorax*, **40**, 85–90.

GOSLIN, R.H., SKARIN, A.T. & ZAMCHECK, N. (1981). Carcinoembryonic antigen. A useful monitor of therapy of small cell lung cancer. *J. Am. Med. Assoc.*, **246**, 2173–2176.

HANSEN, M., HAMMER, M. & HUMMER, L. (1980). ACTH, ADH and Calcitonin as markers of response and relapse in small-cell carcinoma of the lung. *Cancer*, **46**, 2062–2067.

HAVEMANN, K., HOLLE, R. & GROPP, C. (1985). Prospective multicenter study of hormone markers in small cell lung cancer. *Recent Results Cancer Res.*, **99**, 194–208.

JAQUES, G., BEPLER, G., HOLLE, R., WOLF, M., HANNICH, T., GROPP, C. & HAVEMANN, K. (1988). Prognostic value of pretreatment carcinoembryonic antigen, neuron-specific enolase, and creatine kinase-BB levels in sera of patients with small cell lung cancer. *Cancer*, **62**, 125–34.

JORGENSEN, L.G., HANSEN, H.H. & COOPER, E.H. (1989). Neuron specific enolase, carcinoembryonic antigen and lactate dehydrogenase as indicators of disease activity in small cell lung cancer. *Eur. J. Cancer Clin. Oncol*,. **25**, 123–128.

JORGENSEN, L.G., OSTERLIND, K., HANSEN, H.H. & COOPER, E.H. (1988). The prognostic influence of serum neuron specific enolase in small cell lung cancer. *Br. J. Cancer*, **58**, 805–807.

KAPLAN, E.L. & MEIER, P. (1958). Nonparametric estimation from incomplete observations. *J. Am. Statist. Assoc.*, **53**, 457–481.

KRISCHKE, W., NIEDERLE, N., SCHUTTE, J., PFEIFFER, R. & HIRCHE, H. (1988). Is there any clinical relevance of serial determinations of serum carcinoembryonic antigen in small cell lung cancer patients? *Cancer*, **62**, 1348–1354.

LABERGE, F., FRITSCHE, H.A., UMSAWASDI, T., CARR, D.T., WELCH, S., MURPHY, W.K., CHIUTEN, D.F., DHINGRA, H.M., FARHA, P., SPITZER, G. & VALDIVIESO, M. (1987). Use of carcinoembryonic antigen in small cell lung cancer. Prognostic value and relation to the clinical course. *Cancer*, **59**, 2047–2052.

LOKICH, J.J. (1982). Plasma CEA levels in small cell lung cancer. *Cancer*, **50**, 2154–2156.

NORTH, W.G., WARE, J., MAURER, L.H., CHAHINIAN, A.P. & PERRY, M. (1988). Neurophysins as tumor markers for small cell carcinoma of the lung. *Cancer*, **62**, 1343–1347.

O'CONNOR, D.T. & DEFTOS, L.J. (1986). Secretion of chromogranin A by peptide-producing endocrine neoplasms. *N. Engl. J. Med.*, **314**, 1145-1151.

SCULIER, J.P., FELD, R., EVANS, W.K., SHEPHERD, F.A., DEBOER, G., MALKIN, D.G. & MALKIN, A. (1985). Carcinoembryonic antigen: a useful prognostic marker in small-cell lung cancer. *J. Clin. Oncol.*, **3**, 1349-1354.

SHINKAI, T., SAIJO, N., TOMINAGA, K., EGUCHI, K., SHIMIZU, E., SASAKI, Y., FUJITA, J., FUTAMI, H., OHKURA, H. & SUEMASU, K. (1986). Serial plasma carcinoembryonic antigen measurement for monitoring patients with advanced lung cancer during chemotherapy. *Cancer*, **57**, 1318-1323.

SLEVIN, M.L., CLARK, P.I., JOEL, S.P., MALIK, S., OSBORNE, R.J., GREGORY, W.M., LOWE, D.G., REZNEK, R.H. & WRIGLEY, P.F.M. (1989). A randomized trial to evaluate the effect of schedule on the activity of etoposide in small-cell lung cancer. *J. Clin. Oncol.*, **7**, 1333-1340.

SOBOL, R.E., O'CONNOR, D.T., ADDISON, J., SUCHOCKI, K., ROYSTON, I. & DEFTOS, L.J. (1986). Elevated serum chromogranin A concentrations in small-cell lung carcinoma. *Ann. Intern. Med.*, **105**, 698-700.

SOUHAMI, R.L. & LAW, K. (1990). Longevity in small cell lung cancer. A report to the Lung Cancer Subcommittee of the United Kingdom Coordinating Committee for Cancer Research. *Br. J. Cancer*, **61**, 584-589.

WAALKES, T.P., ABELOFF, M.D., ETTINGER, D.S., WOO, K.B., GEHRKE, C.W. & KUO, K.C.B.E. (1982). Biological markers and small cell carcinoma of the lung. *Cancer*, **50**, 2457-2464.

WOO, K.B., WAALKES, T.P., ABELOFF, M.D., ETTINGER, D.S., MCNITT, K.L. & GEHRKE, C.W. (1981). Multiple biologic markers in the monitoring of treatment for patients with small cell carcinoma of the lung. *Cancer*, **48**, 1633-1642.